# Diagnostic performance of lactate dehydrogenase (LDH) isoenzymes levels for the severity of COVID-19

**DOI:** 10.5937/jomb0-37234

**Published:** 2023-01-20

**Authors:** Ilkay Ergenc, Emre Capar, Sengel Buket Erturk, Gunel Bahramzade, Fatih Atalah, Derya Kocakaya, Sait Karakurt, Goncagul Haklar, Zekaver Odabasi

**Affiliations:** 1 Marmara University, School of Medicine, Department of Gastroenterology, Istanbul, Turkey; 2 Marmara University, School of Medicine, Department of Internal Medicine, Istanbul, Turkey; 3 Marmara University, School of Medicine, Department of Infectious Disease and Clinical Microbiology, Istanbul, Turkey; 4 Marmara University, School of Medicine, Department of Biochemistry, Istanbul, Turkey; 5 Marmara University, School of Medicine, Department of Pulmonary Medicine, Istanbul, Turkey

**Keywords:** coronavirus, COVID-19, lactate dehydrogenase, LDH isoenzymes, koronavirus, COVID-19, laktat dehidrogenaza, LDH izoenzimi

## Abstract

**Background:**

Lactate dehydrogenase (LDH) levels predict coronavirus disease 2019 (COVID-19) severity. We investigated LDH isoenzyme levels to identify the tissue responsible for serum LDH elevation in patients with COVID-19.

**Methods:**

Hospitalised COVID-19 patients with serum LDH levels exceeding the upper reference limit included. LDH isoenzymes were detected quantitatively on agarose gels. The radiological severity of lung involvement on computed tomography was scored as 0-5 for each lobe (total possible score, 0-25). Disease severity was determined using the World Health Organization (WHO) clinical progression scale.

**Results:**

In total, 111 patients (mean age, 59.96 ± 16.14), including 43 females (38.7%), were enrolled. The serum levels of total LDH and all five LDH isoenzymes were significantly higher in the severe group. The levels of all LDH isoenzymes excluding LDH5 positively correlated with the WHO score. LDH3 levels correlated with chest computed tomography findings (r^2^ = 0.267,* p* = 0.005). On multivariate analysis, LDH3 was an independent risk factor for the deterioration of COVID-19.

**Conclusions:**

LDH3 appears to be an independent risk factor for deterioration in patients with COVID-19. LDH elevation in patients with COVID-19 predominantly resulted from lung, liver and muscle damage.

## Abstract (sr)

**Uvod:**

Nivoi laktat dehidrogenaze (LDH) predviđaju ozbiljnost bolesti korona virusa 2019 (COVID-19). Istraživali smo nivoe LDH izoenzima da bismo identifikovali tkivo odgovorno za povišenje LDH u serumu kod pacijenata sa COVID-19.

**Metode:**

Uključeni su hospitalizovani pacijenti sa COVID-19 sa nivoom LDH u serumu koji prelazi gornju referentnu granicu. LDH izoenzimi su kvantitativno detektovani na agaroznim gelovima. Radiološka težina zahvata pluća na kompjuterizovanoj tomografiji ocenjena je sa 0-5 za svaki režanj (ukupan mogući rezultat, 0-25). Težina bolesti je određena korišćenjem skale kliničke progresije Svetske zdravstvene organizacije (SZO).

## Introduction

Because of a lack of treatment options, coronavirus disease 2019 (COVID-19) has paralyzed health care systems globally. In an effort to predict severity and prognosis, clinicians and researchers have focused on basic laboratory markers to properly identify high-risk patients and reduce the burden on health care systems. Multiple meta-analyses revealed the significance of various laboratory parameters for selecting severe and critical cases and suggested predictive models [1] [2] [3] [4]. Those studies described lactate dehydrogenase (LDH) as an important parameter for predicting disease severity in COVID-19 [3] [4] [5]. A meta-analysis demonstrated that the risk for severe COVID-19 is up to 12-fold higher in patients with elevated serum LDH levels [6].

Lactate dehydrogenase (EC 1.1.1.27; L-lactate: NAD^+^ oxidoreductase; LDH) is a hydrogen transfer enzyme that catalyzes the oxidation of L-lactate to pyruvate with the mediation of NAD^+^ as a hydrogen acceptor. The enzyme catalyzes the final step of glycolysis. It exists in the cytosol of all nucleated cells and erythrocytes [7]. Because it displays a wide tissue and organ distribution, LDH is a highly sensitive but nonspecific marker of tissue damage. LDH contains zinc in a tetrameric structure consisting of different combinations of the active H and M subunits. Five LDH isoenzymes have been identified, and they can be separated electrophoretically according to their subunit composition. LDH1 and LDH2, the most rapidly moving isoenzymes on electrophoresis, are predominant in heart muscle, erythrocytes and the kidneys. Conversely, LDH4 and LDH5 are dominant in the liver and skeletal muscle. LDH3 is most commonly found in lung tissue [7] [8]. LDH isoenzymes can be quantified measured after electrophoresis separation to optimize diagnosis and specify the tissue of origin. In this study, we investigated LDH isoenzyme levels to identify the tissue LDH responsible for serum elevation in patients with COVID-19.

## Materials and methods

### Patients

The study was conducted prospectively in a COVID-19 referral centre (Marmara University Hospital) from 4 May 2021 to 24 July 2021. Consecutive hospitalized patients with COVID-19, as confirmed via combined nasopharyngeal and oropharyngeal SARS-COV-2 reverse transcrip tasepoly merase chain reaction, were screened for serum LDH level elevation. Patients with a history of chronic liver, lung and muscle disease, as well as those with malignancy and haemolytic anaemia, were excluded. Patients with serum LDH levels exceeding the upper reference value were included. The patients’ clinical data, including symptoms, co-morbid diseases, length of hospital stay, intensive care unit (ICU) admission, World Health Organization (WHO) clinical progression scale, laboratory results, and treatments, were noted. According to the WHO clinical progression scale, patients were categorized into moderate and severe disease groups. The length of hospital stay was categorized as short (1–7 days), intermediate (8–14 days), or long (>14 days).

### Collection of serum samples and laboratory analysis

Blood samples were obtained via venepuncture from each patient after an overnight fast. All laboratory tests were performed in the Biochemistry Laboratory at Marmara University Pendik Education and Research Hospital, Istanbul, Turkey. Blood samples were centrifuged at 1500 rpm for 5 min. All laboratory tests excluding LDH electrophoresis were performed on the same day. Serum samples for electrophoresis were separated within two hours of collection and stored at −80°C.

Blood samples for cell counts, serum markers and coagulation markers were analyzed within three hours of collection. Complete blood counts were measured in EDTA samples using a Unicel DxH800 Coulter Cell Analyzer (Beckman Coulter, California, USA). Total LDH, alanine aminotransferase (ALT), aspartate aminotransferase (AST), γ-glutamyl transferase (GGT), creatine kinase (CK), total bilirubin and direct bilirubin levels were analyzed spectrophotometrically using an AU 680 instrument (Beckman Coulter). Ferritin levels were measured via a two-site immunoenzymatic assay using Access Analyzer (Beck man Coulter). Troponin T levels were determined using an electrochemiluminescence immunoassay (Modular Analytics E170, Roche Diagnostics, Germany). D-dimer content was quantitated using an immunoturbidimetric assay in venous plasma (in 3.2% sodium citrate) with STA Compact (Diagnostica Stago, France).

Frozen serum samples were used to analyze isoenzyme levels. Haemolysed samples were excluded. An Interlab G26 electrophoresis kit was used for the quantitative detection of isoenzymes. LDH isoenzymes were separated on an agarose gel according to an isoelectric gradient at pH 8. After electrophoretic migration, the gel was treated with stain reagent (10.0 nmol/L NAD, 300 nmol/L lactate, 11.1 nmol/L NBT and 0.375 nmol/L PMS) and incubated at 45°C for 30 min. After incubation, the gel was treated with destain reagent (concentrated (5%) acetic acid) and dried for 20 min at 70°C. The plate was scanned within two hours.

### Reference intervals and electrophoresis patterns

Because there are no specific cut-off values for normal serum LDH isoenzyme levels, both the expected proportions of isoenzymes and their electrophoretic patterns were used to interpret the results. The expected normal proportions of LDH isoenzymes for healthy controls (expressed as a percent of total LDH) are 20%–30%, 30%–40%, 20%–25%, 7%–15% and 5%–12% for LDH1, LDH2, LDH3, LDH4 and LDH5, respectively (Table 1). Normal serum LDH isoenzyme levels decrease in the order of LDH2 >LDH1 > LDH3 > LDH4 > LDH5.

**Table 1:** Expected and calculated cut-offs of lactate dehydrogenase (LDH) isoenzyme levels. LDH, lactate dehydrogenase; ROC, receiver operating characteristic

Isoenzymes	Peptide structures	Expected proportion	Discriminative cut-off on ROC analysis
LDH1	HHHH (H4)	20%–30%	68.8 (27.7%)
LDH2	HHHM (H3M)	30%–40%	117.65 (47.4%)
LDH3	HHMM (H2M2)	20%–25%	81.72 (32.9%)
LDH4	HMMM (HM3)	7% –15%	40.85 (%16.4%)
LDH5	MMMM (M4)	5%–12%	46.95 (18.9%)

The definitions of elevated serum LDH patterns in several diseases are as follows:

• *Isomorphic pattern*: Increased total LDH levels with normal proportions of isoenzymes

• *Lung pattern*: Proportional increases of LDH3, LDH4 and LDH5 levels

*Tombstone pattern*: Relatively similar increases in the levels of all isoenzymes.

### Radiological assessment

Chest scans were obtained using the same spiral computed tomography (CT) machine (Siemens Sensation 40, Siemens Medical Solutions, Erlangen, Germany) with a slice thickness of 2 mm. CT was performed on the day of admission in 82 patients, 1 week before admission (median, 5 days) in 24 patients and during the second or third week of hospitalization in five patients. All CT images were reviewed by two pulmonologists with 10 and 20 years of experience, respectively, in radiology. Each lobe was assigned a score as follows: 0, 0% involvement; 1, less than 5% involvement; 2, 5%–25% involvement; 3, 26%–49% involvement; 4, 50%–75% involvement; and 5, greater than 75% involvement. Thus, the score ranged 0–5 for each lobe, and the total possible score ranged 0–25 [9]. Quantitative analysis showed high consistency between two pulmonologists (intragroup correlation coefficient for the total score was 0.946 for all patients). Both pulmonologists reviewed CT images with incompatible scores in the same session, and the scores that were decided upon reaching consensus were included in the analysis.

### Statistical analysis

The Shapiro–Wilk test was used to assess whether the variables followed a normal distribution. Descriptive statistics are presented as the frequency (%), median (range), median (interquartile range), or mean ± standard deviation. According to the normality test results, the Mann–Whitney U test, in dependent-samples Student’s t-test, Kruskal–Wallis test with Dunn–Bonferroni correction for pairwise comparisons or one-way analysis of variance was performed to compare groups. Categorical variables were compared using the chi-squared test, Fisher’s exact test or Fisher–Freeman–Halton test. Spearman’s correlation coefficient was used to examine correlations. To estimate the cut-off, sensitivity and specificity of LDH isoenzymes for predicting ICU admission and death, receiver operator characteristic (ROC) curve analysis was performed. The determination of risk factors for ICU admission and death was performed via binary logistic regression using the forward stepwise (likelihood ratio) method. SPSS (released 2019, IBM SPSS Statistics for Windows, Version 26.0., IBM, Armonk, NY, USA) and MedCalc Statistical Software version 16.4.3 (MedCalc Software by, Ostend, Belgium; https://www.medcalc.org; 2016) were used for analysis. Significance was indicated by *p*< 0.05.

*Ethical approval*: The study was conducted following the ethical standards of the responsible committee on human experimentation and the Declaration of Helsinki of 1975, as revised in 2008, and the local ethics committee of Marmara University School of Medicine approved the study protocol (Date: 20-05-2020, Number: 09.2020.560).

## Results

### Demographic, clinical and laboratory findings

The demographic and clinical characteristics of the patients are presented in Table 2. In total, 123 hospitalized patients were included in the study. Five patients were excluded after receiving a diagnosis of acute myocardial infarctions (n = 2), ischaemic hepatitis (n = 2) or metastatic prostate cancer (n = 1) during hospitalization, and seven patients with haemolysed serum samples were also excluded. The remaining 111 patients were included in the final analysis. The mean patient age was 59.96 ± 16.14 years, and 38.7% (n = 43) of the patients were female. The median length of hospitalization was 13 days (4–115).

**Table 2:** Demographic and clinical characteristics of the patients. WHO, World Health Organization; ICU, intensive care unit; DM, diabetes mellitus, HT, hypertension; CAD, coronary artery disease; COPD, chronic obstructive lung disease; CLD, chronic liver disease; CKD, chronic kidney disease; AST, aspartate aminotransferase; ALT, alanine aminotransferase; CK, creatine kinase; LDH, lactate dehydrogenase; CT, computed tomography. ^†^ Moderate disease defined as a WHO score of ≤5. ^‡^Severe disease is defined as a WHO score of ≥6 (ICU admission or death).

	Moderate disease^†^<br>(n = 65)	Severe disease^‡^<br>(n = 46)	All patients<br>(n = 111)	*p*-value
Age	57.54 ± 16.26	63.39 ± 15.50	59.96 ± 16.14	0.059
Gender (female)	27 (41.54%)	16 (34.78%)	43 (38.74%)	0.472
Symptoms at admission<br>Respiratory symptoms<br>Gastrointestinal symptoms<br>Myalgia	<br>40 (61.54%)<br>8 (12.31%)<br>13 (20.00%)	<br>38 (82.61%) <br>3 (6.52%) <br>3 (6.52%)	<br>78 (70.27%) <br>11 (9.91%) <br>16 (14.41%)	<br>0.017 <br>0.357 <br>0.046
Length of hospital stay (days)	11 (4–37)	19 (5–115)	13 (4–115)	<0.001
Co-morbidities<br>DM (yes)<br>HT (yes)<br>CAD (yes)<br>COPD (yes)<br>CKD (yes)	<br>11 (16.92%)<br>23 (35.38%)<br>6 (9.23%)<br>6 (9.23%)<br>3 (4.61%)	<br>16 (34.78%)<br>22 (47.83%)<br>10 (21.74%)<br>2 (4.35%)<br>7 (15.22%)	<br>27 (24.32%)<br>45 (40.54%)<br>16 (14.41%)<br>8 (7.21%)<br>10 (9.01)	<br>0.031<br>0.188<br>0.065<br>0.466<br>0.089
Laboratory results on the test day<br>LDH (U/L)<br>Lymphocyte count (10^3^/mL)<br>Lymphocyte percentage (%)<br>AST (U/L)<br>ALT (U/L)<br>CK (U/L)<br>Troponin I (ng/L)<br>Ferritin (mg/L)<br>Total bilirubin (mg/dL)<br>Direct bilirubin (mg/dL)	<br>311 (243–624)<br>1100 (300–3000)<br>19 (2.80–50)<br>36 (14–215)<br>30 (10–218)<br>74 (13–2031)<br>9.35 (3–589) *n*:42<br>370.50 (10–1850) *n*:58<br>0.60 (0.27–1.60)<br>0.10 (0.01–0.40)	<br>411.50 (281–881)<br>900 (100–2000)<br>14.50 (1.40–86)<br>57.50 (19–298)<br>45.50 (8–269)<br>79.50 (14–1143)<br>16 (3–500) n:45<br>461.50 (10–3689)<br>0.60 (0.18–3.37)<br>0.10 (0.01–2.44)	<br>350 (243–881)<br>1000 (100–3000)<br>17 (1.4–86)<br>39 (14–298)<br>36 (8–269)<br>75 (13–2031)<br>12 (3–589)<br>388 (10–3689)<br>0.6 (0.18–3.37)<br>0.1 (0.01–2.44)	<br><0.001<br>0.004<br>0.002<br><0.001<br>0.071<br>0.720<br>0.014<br>0.076<br>0.891
LDH isoenzymes<br>LDH1 (U/L)<br>LDH2 (U/L)<br>LDH3 (U/L)<br>LDH4 (U/L)<br>LDH5 (U/L)	<br>63.42 (41.65–141.65)<br>106.80 (39.53–219.45)<br>70.18 (29.07–151.63)<br>31.84 (12.97–71.20)<br>39.30 (13.54–217.55)	<br>83.25 (37.65–189.06)<br>142.09 (73.75–292.41)<br>91.29 (53.39–246.68)<br>43.24 (15.81–117.11)<br>48.92 (10.22–191.27)	<br>70.22 (37.65–189.06)<br>117.42 (39.53–292.41)<br>81.34 (29.07–246.68)<br>34.51 (12.97–117.11)<br>43 (10.22–217.55)	<br><0.001<br><0.001<br><0.001<br><0.001<br>0.024
Chest CT score<br>(0–25)	9 (1–21)	15 (5–25)	11 (1–25)	<0.001

According to the WHO scale, patients were categorized as having moderate and severe disease. There were no significant differences in age and gender between the moderate and severe groups (*p* = 0.059 and *p* = 0.472, respectively). Respiratory symptoms on admission were more frequently higher in the severe group (*p* = 0.017), whereas myalgia was more common in the moderate group (*p* = 0.046). The length of hospital stay was longer in the severe group. Among co-morbidities, only the incidence of diabetes mellitus was significantly different between the groups, being more common in patients with severe COVID-19 (*p* = 0.031). Chest CT scores were significantly more common in the severe group (15 vs. 9, *p*< 0.001). Troponin I and CK levels on admission were significantly higher, lymphocyte count and lymphocyte percentage were significantly lower in the severe group severe on admission (*p* = 0.003). AST and ALT levels on admission did not differbetween the groups. As expected, patients in the severe group more commonly used tocilizumab and methylprednisolone (*p*< 0.001 and *p* = 0.016, respectively). Conversely, hydroxychloroquine, azithro mycin, enoxaparin and convalescent plasma treatment rates were similar between the groups.

### LDH isoenzyme electrophoresis

**Figure 1:**
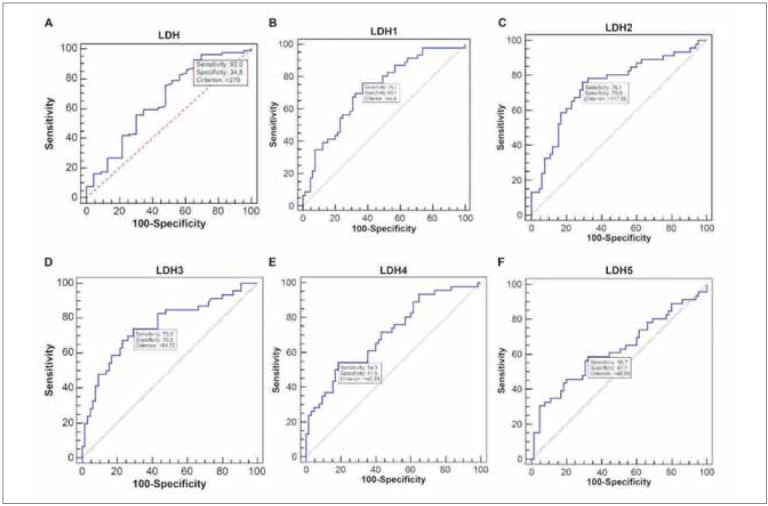
Receiver operating curve analysis of the cut-offs for total lactate dehydrogenase (LDH) and LDH isoenzyme levels for discriminating disease severity. Total LDH levels showed the best area under the curve. The cut-off value of total LDH levels for predicting disease severity was >345U/L (AUC = 0.801, sensitivity = 82.7%, specificity = 69.2%). The cut-off values for LDH1, LDH2, LDH3, LDH4 and LDH5 were >68.8, >117.65, >81.72, >40.85, and >46.95 U/L, respectively.<br>(AUC, area under the curve)

LDH isoenzyme levels increased in the order of LDH2 > LDH3 > LDH1 > LDH5 > LDH4. The proportions of LDH3 and LDH5 levels were higher than expected. The mean proportions of LDH1, LDH2, LDH3, LDH4 and LDH5 were 20.58%, 33.42%, 22.53%, 10.00% and 13.63%, respectively. Figure 1 presents the results of the ROC analysis.

Among the 111 patients enrolled in the final analysis, the proportions of LDH1, LDH2, LDH3, LDH4 and LDH5 were higher than expected in 5.4 (n = 6), 10.8 (n = 12), 56.8 (n = 63), 4.5 (n = 5) and 26.1% (n = 29) of patients, respectively. The serum levels of total LDH and all LDH isoenzymes were significantly higher in the severe group (*p*< 0.001, *p*< 0.001, *p*< 0.001, *p*< 0.001 and *p* = 0.024, respectively). Regarding the electrophoresis patterns, 43.2 (n = 48), 46.8 (n = 52) and 7.2% (n = 8) of patients had isomorphic, lung and tombstone patterns, respectively, whereas the pattern could not be classified for three patients.

Serum ALT, AST and CK levels were significantly different among the electrophoresis patterns (*p* = 0.011, *p* = 0.019 and *p* = 0.006, respectively). In particular, CK levels differed between the isomorphic and lungs pattern and between the tombstone and lung patterns (*p* = 0.008 and *p* = 0.046, respectively). ALT levels significantly differed between the isomorphic and lung patterns and between the tombstone and isomorphic patterns (*p* = 0.008 and *p* = 0.046, respectively). AST levels significantly differed between the isomorphic and lung patterns (*p* = 0.010). The WHO severity scale, CT scores, lymphocyte count and D-dimer level did not differ among the electrophoresis patterns (Table 3).

**Table 3:** LDH patterns on electrophoresis. LDH, lactate dehydrogenase; WHO, World Health Organization; AST, aspartate aminotransferase; ALT, alanine aminotransferase; CK, creatine kinase

	Isomorphic	Tombstone	Lung	Other	*p*-value		
WHO severity<br>scale	5 (2–10)<br>(n = 48)	5.50 (5–10)<br>(n = 8)	5 (2–10)<br>(n = 52)	5 (2–6) (n = 3)	0.675		
CT score	11.48 ± 6.13<br>(n = 46)	13.13 ± 9.37<br>(n = 8)	11.94 ± 5.96<br>(n = 51)	8.33 ± 3.21<br>(n = 3)	0.705		
Lymphocyte<br>count<br>(103/mL)	1100<br>(500–3000)<br>(n = 48)	1100<br>(600–1700)<br>(n = 8)	1000<br>(100–2400)<br>(n = 52)	1000<br>(500–1900)<br>(n = 3)	0.500		
CK (U/L)	57<br>(14–1143)<br>(n = 45)	47.50<br>(13–280)<br>(n = 8)	103<br>(14–2031)<br>(n = 50)	176<br>(87–300)<br>(n = 3)	0.011	Isomorphic/<br>lung	0.008
						Tombstone/<br>lung	0.046
ALT (U/L)	24.50<br>(10–228)<br>(n = 48)	84<br>(26–168)<br>(n = 8)	41.50<br>(8–218)<br>(n = 52)	38<br>(36–269)<br>(n = 3)	0.019	Isomorphic/<br>lungs	0.040
						Isomorphic/<br>tombstone	0.009
AST (U/L)	36<br>(15–112)<br>(n = 48)	78<br>(17–298)<br>(n = 8)	56<br>(14–215)<br>(n = 51)	43<br>(41–167)<br>(n = 3)	0.006	Isomorphic/<br>lung	0.010

### ROC analysis

**Figure 2:**
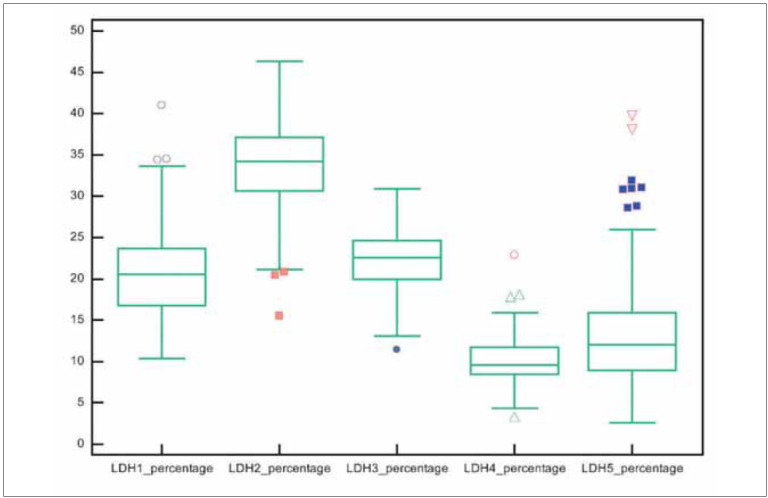
A boxplot of LDH isoenzymes on electrophoresis. The mean proportions of LDH1, LDH2, LDH3, LDH4 and LDH5 were 20.58%, 33.42%, 22.53%, 10.00% and 13.63%, respectively. The proportions of LDH3 and LDH5 were higher than expected. (LDH, lactate dehydrogenase)

ROC analyses were performed to assess the discriminative performance and determine the cut-offs of total LDH and isoenzyme levels for predicting severe COVID-19. All isoenzymes except LDH5 had useful accuracy with AUC between 0.7–0.9 [10]. The cut-offs for LDH1, LDH2, LDH3, LDH4 and LDH5 according to ROC analysis and the diagnostic ability of LDH isoenzyme levels to predict severity are summarised in Table 4. Total LDH levels had the best area under the curve (AUC, Figure 2). The cut-off for total LDH levels for predicting severe disease was >345U/L (AUC = 0.801, sensitivity = 82.7%, specificity = 69.2%).

**Table 4:** Receiver Operating Characteristic (ROC) Curve Analysis and Diagnostic Ability of Evaluated LDH isoenzyme levels Tests to Predict Severity in Studied Coronavirus Disease 2019 (COVID-19) Patients Using the Best Cut off. LDH, lactate dehydrogenase; CI, confidence interval

Test	Test	Selected Cut	Specificity	Sensitivity
LDH1	0.721	68.8 U/L	63.1%	76.1%
LDH2	0.741	117.65 U/L	70.8%	76.1%
LDH3	0.755	81.72 U/L	70.8%	73.9%
LDH4	0.708	40.85 U/L	81.5%	54.3%
LDH5	0.626	46.95 U/L	67.7%	58.7%

### Chest CT findings and LDH

All but five patients underwent CT before admission to the hospital. The median time between CT and LDH electrophoresis was 5 days (0–18). LDH3 levels and chest CT scores were weakly correlated (r^2^ = 0.267, *p* = 0.005). Combined levels of LDH3–5 (lung pattern) were weakly correlated with chest CT scores findings (r^2^ = 0.256, *p* = 0.008). Chest CT scores were not significantly correlated with the LDH3 percentage (r^2^ = −0.003, *p* = 0.977) and LDH3–5 (r^2^ = −0.004, *p* = 0.966) on electrophoresis. CT scores did not differ between patients with normal and elevated LDH3 levels.

### Relationship between CK and LDH levels

LDH4 (r^2^ = 0.293, *p* = 0.002), LDH5 (r^2^ = 0.232, (*p* = 0.014) and LDH4–5 (r^2^ = 0.275, *p* = 0.004) levels were weakly correlated with CK levels. Patients with high CK levels had higher median LDH5 levels (39.7 U/L vs. 62.9 U/L, *p* = 0.004). Patients with a higher proportion of LDH5 (>12%) had higher median serum levels of AST, ALT and CK (Table 5).

**Table 5:** Comparison of transaminase and creatine kinase levels between patients with normal and elevated LDH5 levels. CK, creatine kinase; ALT, alanine aminotransferase; AST, aspartate aminotransferase

	Normal LDH5<br>on electrophoresis<br>(n = 52)	LDH5 dominance<br>on electrophoresis<br>(n = 59)	p-value
CK (U/L)	37 (14–185)	81 (17–298)<br>(n = 58)	<0.001
ALT (U/L)	29.50 (8–228)	85 (17–269)<br>(n = 59)	<0.001
AST (U/L)	37 (14–185)	81 (17–298)<br>(n = 29)	<0.001

### WHO scale

Patients with severe disease had higher median levels of LDH3–5 (133.0 U/L (108.5–184.9) vs. 101.5 U/L (87.3–121.3) *p*< 0.001). Similarly, patients with high LDH4 and LDH4–5 levels had a higher WHO score than those with normal levels (6 vs. 5, *p* = 0.001). All LDH isoenzymes excluding LDH5 had a positive correlation with the WHO score. Table 6 presents the correlation between LDH isoenzyme levels and the WHO scale. Total LDH levels significantly differed according to the WHO scale for clinical improvement (*p*< 0.001). However, after Bonferroni correction, no difference was found (p > 0.05).

**Table 6:** Associations between LDH isoenzyme levels and the World Health Organization scale for clinical improvement. WHO scale for clinical improvement is presented with numbers for the stated pairwise comparisons. LDH, lactate dehydrogenase; WHO, World Health Organization

	Ambulatory mild disease<br>(WHO score = 2)<br>(n = 10)	Moderate disease<br>(WHO score = 4–5)<br>(n = 55)	Severe disease<br>(WHO score = 6–9)<br>(n = 31)	Death<br>(WHO score = 10)<br>(n = 15)	p-value
LDH (U/L)	308.50 (250–416)	311 (243–624)	406 (281–649)	413 (294–881)	0.000
LDH1 (U/L)	69.74 (50.69–133.13)	62.99 (41.65–141.65)	83.04 (56.20–145.10)	84.25 (37.65–189.06)	0.001
LDH2 (U/L)	110.97 (39.53–157.25)	106.020 (55.49–219.45)	136.52 (81.81–221.91)	148.80 (73.75–292.41)	0.000
LDH3 (U/L)	68.05 (29.07–89.40)	70.72 (39.37–151.63)	90.67 (53.39–181.81)	91.68 (73.13–246.68)	0.000
LDH4 (U/L)	26.53 (18.96–39.66)	33.40 (12.97–71.20)	41.40 (15.81–117.11)	45.84 (24.11–82.90)	0.001

### Multivariate analysis

The LDH level (odds ratio (OR) = 1.248 (95% confidence interval (CI) = 1.101–1.445), *p* = 0.001), chest CT score (OR = 0.995 (95% CI = 0.992–0.997), *p* = 0.000) and lymphocyte count (OR = 1.012 (95% CI = 1.005–1.019), *p* = 0.001) were independent factors for severe disease in multivariate analysis. When all LDH isoenzymes levels were tested in multivariate analysis, the chest CT score, lymphopenia and LDH3 levels (OR = 1.049 (95% CI = 1.019–1.080), *p* = 0.00) remained predictive of severe disease (Table 7).

**Table 7:** Univariate and Multivariate Logistic Regression Analyses for Predictors of severity of Coronavirus Disease 2019 (COVID-19) Patients. CT, computed tomography; LDH, lactate dehydrogenase; AST, aspartate aminotransferase; ALT, alanine aminotransferase; CK, creatine kinase

	Univariate analysis	Multivariate analysis		
	p-value	Odds ratio	95% confidence interval	p-value
Lymphocyte count	0.001	0.995	0.992–0.997	0.000
Chest CT score	<0.001	1.272	1.118–1.447	0.000
LDH1	0.001			
LDH2	<0.001			
LDH3	<0.001	1.049	1.019–1.080	0.001
LDH4	<0.001			
LDH5	0.024			
Age	0.059			
Gender (female)	0.472			
Diabetes mellitus	0.031			
Hypertension	0.188			
AST	0.095			
ALT	0.409			
CK	0.085			
Respiratory symptoms	0.017			
Gastrointestinal symptoms	0.357			
Myalgia	0.046			

## Discussion

This study investigated LDH isoenzyme levels to identify the tissue responsible for serum LDH level elevation in patients with COVID-19. More than half of the patients had increased LDH3 levels, and nearly one-third had increased LDH5 levels. Moreover, when the mean concentrations of all isoenzymes were analyzed, their levels increased in the order of LDH2 > LDH3 > LDH1 > LDH5 > LDH4.

LDH is a hydrogen transfer enzyme present in the cytoplasm of almost all cells. Upon tissue damage, LDH is released into the bloodstream. Thus, serum LDH elevation generally indicates tissue damage. Although serum LDH levels help predict the severity of COVID-19, the mechanism behind this relationship and the tissue responsible for serum LDH elevation remain uncertain. Under physiological conditions, various tissues have approximately 500-1500-fold higher LDH levels than serum. Thus, even a small amount of tissue damage leads to significantly elevated serum LDH levels [7]. Assessment of other organ-specific damage markers cannot reveal the tissue of origin in patients with elevated LDH levels.

LDH isoenzymes can improve diagnostic accuracy and identify the tissue of origin in certain diseases, especially pulmonary pathologies [11]. Most patients infected with SARS-COV-2 usually have a mild-to-moderate illness, but approximately 5% of patients develop the critical disease with pneumonia and respiratory failure [12]. In this study, the change in the proportion of LDH-3 was most prominent. LDH3 levels are highest in lung tissue [7]. Studies on pulmonary embolism, *Pneumocystis carinii *pneumonia and mycoplasma pneumonia confirmed that LDH3 is useful for monitoring pulmonary inflammation in both vascular and infectious pathologies [13] [14] [15].

In addition to the predominance of LDH, nearly half of the patients presented with the lung pattern on electrophoresis. The lung pattern is characterized by proportional increases in LDH3, LDH4 and LDH5 levels. In animal models, both acute pulmonary embolism and acute immunological lung damage result in increased serum LDH3 activity in the first 24–48 h [15] [16]. However, during the sub-acute period in patients with pulmonary embolism in clinical studies, elevated serum LDH levels with lung patterns were observed as opposed to increased LDH3 levels alone [14] [17]. During chronic rejection following lung transplantation, the lung pattern of elevated serum LDH activity is also detected [18]. Acute lung injury has been suggested to result in increased LDH3 activity via injured alveolar-capillary and endothelial cells [11] [10]. Although LDH3 is most commonly found in lung tissues, the distribution of LDH isoenzymes in lung tissues is estimated to be 10% LDH1, 20% LDH2, 30% LDH3, 25% LDH4 and 15% LDH5 [8]. Thus, there appears to be a timerelated effect in LDH isoenzyme activity in lung injury.

LDH3 levels were weakly correlated with radiological severity on chest CT in this study. This may have resulted from the time gap between CT and the LDH test. Nearly all patients underwent CT before admission to the hospital, whereas serum LDH elevation occurred subsequently. This may be another reason for this weak correlation.

The slow-acting isoenzymes LDH4 and LDH5 are predominant in the liver and skeletal muscle [7]. In our study, LDH5 levels were increased to a lesser extent, and these changes were correlated with those of liver and muscle enzymes. Patients with an increased proportion of LDH5 on electrophoresis had significantly higher median CK, AST and ALT levels. Almost half of the patients with COVID-19 have elevated liver enzyme levels. Those with increased transaminase levels on admission are more likely to develop hypoxia or severe inflammation [19].

Furthermore, patients with elevated liver enzyme levels on admission or follow-up have an increased risk of life-threatening complications, ICU admission and death [20] [21]. Some authors reported increased CK levels and suggested associations of its elevation with poor prognosis in the course of COVID-19 [2] [22] [23]. Thus, the liver and muscle tissue might be other sources of serum LDH. Moreover, morbidity and mortality risks attributed to serum LDH elevation may also arise from these tissues, in addition to lung damage.

The optimal serum total LDH cut-off for predicting severe disease was 345 U/L in the present study. In a large case study from Italy, the researchers found that the optimal serum total LDH cut-off for predicting intensive treatment was 425 U/L [24]. Unlike this study, we only included patients with elevated serum total LDH levels. The major strength of our study was using the WHO scale to stratify disease severity. This study was thus the first to compare the discriminative power of LDH isoenzymes with total LDH for discriminating severe diseases.

All isoenzyme levels were higher in patients with severe COVID-19. On individual ROC analysis, serum total LDH had the best AUC. Although individual LDH isoenzymes typically predominate in certain tissues, such as LDH in the lungs or LDH1 in the heart, most tissues produce all LDH isoenzymes. Thus, even in specific tissue injuries, all isoenzyme levels rise with the expected predominant isoenzyme. This might explain the greater predictive utility of total LDH than the isoenzymes.

On multivariate analysis, LDH3 was an independent risk factor for the deterioration of COVID-19, together with the chest CT score and lymphocyte count. Many tissues contribute to the elevation of serum LDH levels, but the predictive utility of LDH3 for severity indicates that the increase in serum LDH levels was attributable to lung injury.

In conclusion, although the increase in total serum LDH levels is rather non-specific, this study found that LDH elevation in patients with COVID-19 was predominantly attributable to lung, liver and muscle damage. These organ systems should specifically be examined to note elevation in the levels of LDH in patients with severe COVID-19. No individual LDH isoenzyme had better predictive utility than total LDH for discriminating COVID-19 severity.

*Key messages*: LDH elevation in patients with COVID-19 is predominantly attributable to lung, liver and muscle damage.

LDH3 levels correlate with chest computed tomography findings and are an independent risk factor for the deterioration of COVID-19.

The predictive utility of LDH3 for severity indicates that the prognostic role of increased serum LDH levels is attributable to lung injury.

*Institutional Review Board Statement*. The study was conducted following the ethical standards of the responsible committee on human experimentation and the Declaration of Helsinki of 1975, as revised in 2008, and the local ethics committee of Marmara University School of Medicine approved the study protocol (Date: 20-05-2020, Number: 09.2020.560).

## Dodatak

### Acknowledgments

The authors thank Ozlem Toluk, Ph.D. student, Department of Biostatistics, Uluda University Institute of Health Sciences, Bursa, Turkey, for assisting them with the statistical review of this study.

### Research funding

None declared.

### Author contributions

Conception and design of the study: IE and ZO

Acquisition of the data: IE, EC, BES, FA, SK and DK

Analysis and interpretation of data: IE, GB, GH and SK

Critical revisions for important intellectual content: IE, BES, GH, ZO and SK

Final approval of the version to be submitted: IE, EÇ, BES, BH, GH; FA, DK, SK and ZO

### Competing interests

The authors declare no financial conflicts of interest.

### Ethical approval

The study was conducted following the ethical standards of the responsible committee on human experimentation and the Declaration of Helsinki of 1975, as revised in 2008, and the local ethics committee XXXX University approved the study protocol (Date: 20-05-2020, Number: 09.2020.560). Verbal informed consent was obtained from all individuals included in this study.

### Conflict of interest statement

All the authors declare that they have no conflict of interest in this work.
